# “Bad Romance”: Links between Psychological and Physical Aggression and Relationship Functioning in Adolescent Couples

**DOI:** 10.3390/bs5020305

**Published:** 2015-06-09

**Authors:** Inge Seiffge-Krenke, William J. Burk

**Affiliations:** 1 Department of Psychology, University of Mainz, Wallstrasse 3, Mainz, 55112, Germany; 2 Behavioural Science Institute, Radboud University Nijmegen, Montessorilaan 3, Nijmegen, 6525 HR, The Netherlands; E-Mail: w.burk@psych.ru.nl

**Keywords:** romantic relationships, dyadic approach, physical and psychological aggression, person-oriented approach

## Abstract

Assortative mating is an important issue in explaining antisocial, aggressive behavior. It is yet unclear, whether the similarity paradigm fully explains frequent displays of aggression in adolescents’ romantic relationships. In a sample of 194 romantic partner dyads, differences between female and male partners’ reports of aggression (psychological and physical) and different measures of relationship functioning (e.g., jealousy, conflicts, and the affiliative and romantic quality of the relationship) were assessed. A hierarchical cluster analysis identified five distinct subgroups of dyads based on male and female reports of psychological and physical aggression: nonaggressive couples, couples with higher perceived aggressiveness (both physical and psychological) by females, couples with higher aggressiveness perceived by males and mutually aggressive couples. A substantial number of non-aggressive dyads emerged. Of note was the high number of females showing one-sided aggression, which was, however, not countered by their partner. The mutually aggressive couples showed the least adaptive relationship functioning, with a lack of supportive, trusting relationship qualities, high conflict rates and high jealousy. The discussion focuses on the different functions of aggression in these early romantic relations, and the aggravating impact of mutual aggression on relationship functioning and its potential antisocial outcomes.

## 1. Introduction

In the adult relationships literature, intimate partner violence is an important research field. Capaldi *et al.* [1] reviewed 228 studies published in the last three decades. There were considerably more adult studies than adolescent studies, most on non-clinical samples. Seventy-eight percent of the adult studies and 95% of the adolescent studies interviewed individuals only. Hence, there is a dearth of research on adolescent couples. Such aggressive behaviors may appear almost as early as romantic relationships emerge [2]. Studies have shown that physical aggression is relatively stable over time within adolescents’ romantic relationships [3] as well as in adults’ marital relationships [4] and can be linked to antisocial behavior across the life span [5].

The present study was designed to shed light on factors that may contribute to partner selection and the impact of aggression at this formative stage of romantic development. Because aggressive interactions unfold in a dyadic context and involve perpetrators as well as victims, this study set out to examine the links between aggressive interactions and relationship functioning in adolescent couples from the perspectives of both partners. Studies that previously investigated violence in romantic couples adopt a socialization perspective that focuses on how a certain interpersonal contexts can lead to violence and aggression in couples. For men and women, exposure to family violence and abuse, socioeconomic status, stress, particularly acculturation stress was directly related to partner violence [1]. Further, psychopathology [6], and a negative relationship quality [7,8] seem to contribute to aggression in romantic couples. Further, as the 12-year longitudinal study by Shortt *et al.* [9] demonstrates, men’s physical and psychological aggression in their early 20s predicted levels of aggressive interactions years later. Hence, there is an urgent need to understand early contributors of physical and psychological aggression and link them to couple characteristics and relationship characteristics.

We adopted a person-oriented approach to identify distinct subgroups of couples based on dyadic reports of physical and psychological aggression. We further explored differences in dyadic relationship functioning in these subgroups, e.g. how physical and psychological aggression is related to other aspects of relationship functioning, such as duration, romantic feelings, conflicts, and jealousy.

## 2. Adolescents’ Romantic Relationships: A Neglected Cause for Youth Aggression and Violence

Dating partners are often met through or with friends. The mechanisms of influence within the peer group and friendship relations have been less examined. During adolescence, aggressive acts in the peer context such as bullying have received considerable attention in research since the early work of [10]. International studies show that bullying increases with age; boys were frequently the perpetrators, but also three times more victimized than females [11]. In addition, a small number of about 6% involve perpetrators as well as victims. While earlier research characterizes bullies as having socio-emotional and cognitive deficits, recent studies show that bullies exhibit very good socio—emotional capacities which they employ strategically, e. g they are coercive and dominant towards victims and empathetic and helpful to bystanders [12]. Such a bi-strategic behavior of bullies may explain their popularity [13]. Thus, explanations of the causes of aggression need to incorporate the broader developmental context with its participants and the function aggression has in this context.

While females as perpetrators were, until recently, not the focus of aggression research, a recent rise in the arrest rate for violent offenses among girls has sparked the interest in understanding factors associated with girls’ aggression [5]. Intimate relationships were considered as risk and protective factors, because marriage was considered to be protective against antisocial behavior, whereas being involved in a dysfunctional relationship resulted in an increase of antisocial behavior, both in males and females [14]. More specifically, for females, such a “bad romance”, e.g., having an aggressive, delinquent male partner elevated females’ chances of remaining on a trajectory of persistent antisocial behavior. The research by Oudekerk, Burgers, and Reppucci [15] substantiated that adolescent females who paired up with an aggressive partner dated aggressive partners in adulthood and experienced significant continuity in violent behavior between adolescence and adulthood.

The question, why aggressive girls pair up with aggressive males, can be explained by the dyadic similarity paradigm [16], e.g., that nonaggressive adolescents would pair up with similar partners and that aggressive adolescents would pair up with similarly aggressive partners. However, this is only half of the story. As for aggression in other developmental contexts, it is important to consider the development and function of romantic relationships and the quality of aggression.

Research on adolescent romantic relationship often focuses on acts of physical aggression e.g. the intentional use of physical force that could hurt the partner and includes mildly aggressive behavior such as pushing, shoving, or scratching as well as severe violent behaviors such as choking, shaking, slapping, or attacking with weapon [17,18]. Between 14% and 50% of adolescents report at least one act of physical aggression occurring in their relationship within a six month period [19,20]. In the context of the same dating relationships, 15% of the girls and 8% of the boys were persistently aggressive with the same partner [4]. Many researchers have demonstrated the negative effects of physical aggression in adolescent romantic relationships and related it to externalizing problem behavior, depression, drug and alcohol use as well as delinquency [21,22].

Such physical aggressive acts warrants the attention they receive; however, they seldom occur in the absence of concomitant psychological aggression, for example, name calling, verbal attacks, defamatory gossip, exclusion, subtle flirting with another partner in an effort to elicit jealousy, and threatening to end the relationship [4]. Compared to the large body of research on physical aggression both in the romantic and the peer domains, research on psychological aggression in romantic relationships has been relatively scant [23,24]. The few existing studies have shown that psychological aggression is not only relatively common, but especially salient for females. Females, as compared to males, are more bothered by psychological aggression, perceive it to have a greater impact, and spend more time thinking about and discussing it, and, when they are aggressive themselves, tend to use psychological aggression [25]. Psychological aggression in romantic relationships is associated with psychosocial maladjustment, depression, and lower levels of relationship quality [26,27,28].

## 3. Risk Factors for Aggression in Romantic Relationships

Several factors must be considered when trying to explain why aggression may occur between romantic partners in such salient and important relationships during adolescence [29]. Adolescents who report dating aggression often describe conflicts with the partner [24]. A frequent source of conflict concerns how partners allocate their time: As romantic partners become increasingly important, the amount of time spent with best friends usually decreases. In addition, adolescents may have difficulties in differentiating between romantic and platonic friendships [30]. Some adolescents are uncertain about how to act in the romantic relationship [31]. In this context, a further reason for aggressive interactions might be communication deficits and different opinions about sex. Gender differences with respect to the willingness to respond to sexual advances have been found, with females waiting longer than males before having sex and males being more likely to accept a sexual offer than females [32].

Similarly, an individual’s propensity to be jealous in romantic relationships can put him or her at risk for aggressive behavior [33]. Research has shown that highly exclusive adolescent couples characterized by one partner’s jealousy of the other being involved in another relationship are prone to psychological and physical aggression [34]. Also, in young adult couples, high scores in jealousy are associated with a variety of types of aggression [35]. From a developmental perspective, midadolescent romantic relationships can be characterized as being in the affection phase [36], where exclusivity of the couple, high affection, and idealization are typical. During this phase, conflict and jealousy may be particularly high, and the risk for experiencing psychological and physical aggression potentially greater than in earlier or later phases of couple formation.

## 4. Aims of the Study

In order to analyze aggression in adolescent romantic relationships, a dyadic perspective is clearly needed. The majority of studies have approached aggression in romantic relationships by investigating one single source (the adolescents), and mainly retrospectively. Relying on a single source and using retrospective reports has typically yielded lower rates of aggression [21]. Further, relationship factors are overall understudied compared to contextual characteristics and behaviors of partners [1].

The first aim of our study was to examine the quality of romantic relationships in mid adolescent couples. As in other studies [30], we anticipated that males’ and females’ romantic relationships would be characterized by affiliative and romantic qualities, involving physical attraction and trust. In accordance with other studies [37], we also expected conflicts and jealousy to be quite high, as the relationship is still fragile and break-ups may occur, even in the same relationship e.g., on-off relationships [29]. In accordance with other studies on gender differences [17,21,38], we anticipated that females would have higher scores in relationship aggression and lower scores for physical aggression.

The second aim of the study was to identify constellations of romantic partner dyads with distinct patterns of psychological and physical aggression. In Gray and Foshee’s study [19], 66% of adolescent dating aggression was mutual. Therefore, in this study, we chose a dyadic approach to account for the fact that both partners may differently contribute to the escalation of conflicts. Overall, following the dyadic similarity paradigm [16], we expected that nonaggressive adolescents would pair up with similar partners and that aggressive adolescents would pair up with similarly aggressive partners. We expected to identify a substantial proportion of dyads in which both partners reported report low levels of psychological and physical aggression. In contrast, we did not expect to find many dyads in which both partners exhibited high levels of psychological and physical aggression, as we anticipated that such relationships would be prone to dissolution [39]. In addition, following the typology of bullies/perpetrators, and victims in the peer domain [40], we expected to find dyads in which one partner reported high levels of psychological or physical aggression (or both) and the other partner reported low levels for either form of aggression. As can be found in the literature on battering [4], it is possible that one-sided aggression, with males being the aggressor and females the victim, can be generalized to apply to adolescent romantic relationships.

Finally, we also wanted to know if mutually aggressive or one-sided aggressive dyads differed with respect to relationship functioning. In general, we expected that dyads with both partners reporting low levels of aggression would demonstrate better relationship functioning (e.g., fewer conflicts, less jealousy and a more trusting relationship quality) than would dyads with both partners reporting high levels of physical or psychological aggression. In accordance with the literature [41,42], we anticipated that couples reporting high mutual aggression would be characterized by more jealousy, more conflict and more preoccupation, e.g. a romantic quality. Due to the scarcity of research using dyadic reports, it was not possible to put forth clear hypotheses regarding one-sided aggressive dyads.

## 5. Method

### 5.1. Participants and Procedure

The sample consisted of 194 heterosexual romantic dyads consisting of adolescent females (Mage = 16.99 years, *SD* = 1.26) and males (Mage = 18.41 years, *SD* = 2.02). Most of the dyads were composed of two German participants (90%); the 27 non-German participants came from 13 different countries. Seventy-three percent of the females and 67% of the males had been raised in two-parent families. The duration of the romantic relationships ranged from less than one month to more than one year, with 27.8% of relationships lasting less than four months, 32.3% between four and 12 months, and 39.9% of relationships lasting more than one year. Participants were recruited from high schools grades 9 to 12 in Germany. After receiving parental consent, we contacted 760 adolescents who reported being currently involved in a romantic relationship. In the school setting, we gave each participant an envelope with the questionnaires to be completed individually and another envelope with the research instrument to be completed by the partner. The envelopes were coded in order to assure anonymity but to allow the partners to be matched. The participants completed the questionnaires independently in their respective homes and then mailed them to us. We received questionnaires from both partners belonging to 219 dyads (29% of total initial sample). For this study, we excluded some dyads because of incomplete information or because the romantic relationship involved two members of the same sex. Thus, the final sample included 194 heterosexual dyads.

### 5.2. Instruments

*Psychological and physical aggression*. Participants completed the Conflict in Adolescent Dating Relationships Questionnaire [43], which assesses the extent to which individuals implement constructive conflict resolution strategies (negotiation and compromise) or non-constructive strategies (coercion, physical and psychological aggression) when they are angry or upset. For each of the 18 items, responses range from 1 (*definitely would*) to 5 (*definitely would not*). For this study, we constructed two subscales. The first scale encompassed psychological aggression (nine items, e.g., “I don’t talk with him/ her,” “I tease him/her,” “I threaten him/her and say I will terminate the relationship”; alphas were 0.79 for males and 0.83 for females). The second scale encompassed physical aggression (9 items, e.g., “I destroy valuable things of hers/his”, “I throw something at him/her,” “I kick him/her,” “I shake him/her,” “I push him/her,” “I hit him/her,” “I throw something against the wall,”; alphas were 0.81 for males and 0.86 for females).

*Romantic relationship experiences*. The quality of romantic relationships was assessed using the Love Experience Questionnaire (LEQ) designed by Hazan and Shaver [44]. Participants reported their level of agreement with 48 statements about their romantic relationship according to a four-point scale ranging from 1 (*strongly disagree*) to 4 (*strongly agree*). These statements constitute 12 subscales (happiness, friendship, trust, fear of closeness, acceptance, emotional extremes, jealousy, obsessive preoccupation, sexual attraction, desire for union, desire for reciprocation, and love at first sight). In an earlier study [30], exploratory factor analyses (varimax rotation with Kaiser normalization) on the same sample indicated two factors that explained 61% of the variance in females’ reports and 62% of variance in males’ reports. In both analyses, the factor structure demonstrated high factor loadings and low cross-loading between factors (lowest factor loading = 0.69; all cross-loadings less than 0.30). Affiliative experiences included 16 items describing acceptance, friendship, trust, and fear of closeness (reversed). Romantic experiences included 20 items describing obsessive preoccupation, sexual attraction, love at first sight, desire for union, and desire for reciprocation. Internal consistencies (Cronbach’s alphas) for affiliative experiences were 0.76 for females and 0.75 for males; internal consistencies for romantic experiences were 0.88 for females and 0.89 for males.

*Conflict prevalence*. In this study, the conflict prevalence measure described the total number of conflicts identified by each participant from a list of nine conflict topics, pertaining to close friends, the peer group, lack of trust, jealousy, sexuality, lack of communication, lack of support, and different personalities.

*Jealousy*. Jealousy with the romantic partner was assessed by the Friendship Jealousy Questionnaire (FJQ) [45]. The FJQ consists of 30 hypothetical situations, 15 of which involve a best friend, who as a potential interloper might interfere with the friendship and 15 of which involve a potential romantic rival who might interfere in their romantic partnership. We selected romantic jealousy for this study. Adolescents and their romantic partners were asked to rate the level of jealousy they would feel in response to each scenario according to a five-point scale ranging from 1 (*not at all true for me*) to 5 (*really true for me*). Internal consistencies for jealousy with romantic partners were 0.81 for females and 0.83 for males.

## 6. Results

### 6.1. Aggression and Relationship Functioning: Gender Differences and Dyadic Correlations

We initially examined differences between female and male partners’ reports of aggression (psychological and physical), jealousy in the dyad’s relationship, and relationship perceptions (affiliative and romantic). Paired *t* tests were used to determine mean-level differences between male and female reports. The means and standard deviations for females’ and males’ reports of psychological and physical aggression and various aspects of psychological functioning are presented in Table 1. The *t-* tests revealed that females reported more psychological and physical aggression, and more jealousy in their relationship than males.

**Table 1:** Mean-Level Differences between Females’ and Males’ Reports of Aggression and Relationship Functioning.

Measure	Female		Male		
	*M*	*SD*	*M*	*SD*	*t*
**Aggression**					
Psychological	1.94	0.66	1.55	0.52	7.34 *
Physical	1.87	0.65	1.56	0.51	6.09 *
Conflict prevalence	3.64	2.02	3.57	2.23	0.51
Jealousy with partner	3.86	0.90	3.56	1.03	3.72 *
**Relationship perceptions**					
Affiliation	3.38	0.35	3.34	0.38	1.37
Romance	3.18	0.44	3.19	0.44	−0.08

Note: N = 189 dyads. * *p* < 0.01.

Table 2 presents bivariate correlations between female and male reports of aggression and relationship functioning. Associations within reporters (actor correlations) describe links between an individual’s own reports of aggression and relationship functioning. Associations between reporters (partner correlations) describe links between an individual’s own reports of aggression and their partner’s reports of relationship functioning. Psychological and physical aggression was negatively associated with self- and partner’s perceptions of affiliation. Perceptions of romance were negatively associated with self-reports of psychological aggression and self and partner’s report of conflict prevalence. Males’ reports of jealousy were positively correlated with females’ reports of both forms of aggression. These correlations indicate that psychological and physical aggression are linked to self- and partner’s reports of relationship functioning.

**Table 2:** Actor and Partner Correlations between Females’ and Males’ Reports of Aggression and Relationship Functioning.

	Female		Male	
	Psychological	Physical	Psychological	Physical
**Conflict prevalence**				
Actor	0.33 **	0.15 *	0.38 **	0.31 **
Partner	0.26 **	0.08	0.36 **	0.21 **
**Jealousy with partner**				
Actor	0.07	0.03	0.07	0.10
Partner	0.19 **	0.19 **	0.03	−0.03
**Relationship perceptions**				
Affiliation				
Actor	−0.29 **	−0.12	−0.48 **	−0.28 **
Partner	−0.16 *	0.03	−0.29 **	−0.19 **
Romance				
Actor	−0.24 **	0.04	−0.18 *	−0.03
Partner	−0.05	0.07	0.01	0.05

Note: * *p* < 0.05; ** *p* < 0.01.

### 6.2. Types of Romantic Relationships Based on Psychological and Physical Aggression

A hierarchical cluster analysis was used to identify distinct subgroups of dyads based on the standardized scores of male and female reports of psychological and physical aggression. The analysis was performed with the Cluster module of the Sleipner program (version 2.1) [46], which uses an agglomerative clustering algorithm (Ward’s Method) to differentiate clusters by attempting to minimize within-cluster variance and maximize between-cluster variance. The most appropriate number of clusters was determined based on the size and distinctiveness of each cluster and according to Bergman, Magnusson, and El-Khouri’s [47] recommendations. The latter includes cluster solutions explaining approximately two-thirds of the total error sum of squares (indicating that the cluster solution adequately explains the observed data) and within-group homogeneity coefficients (estimated as the average within-cluster Euclidean distances) of less than one.

**Figure 1:**
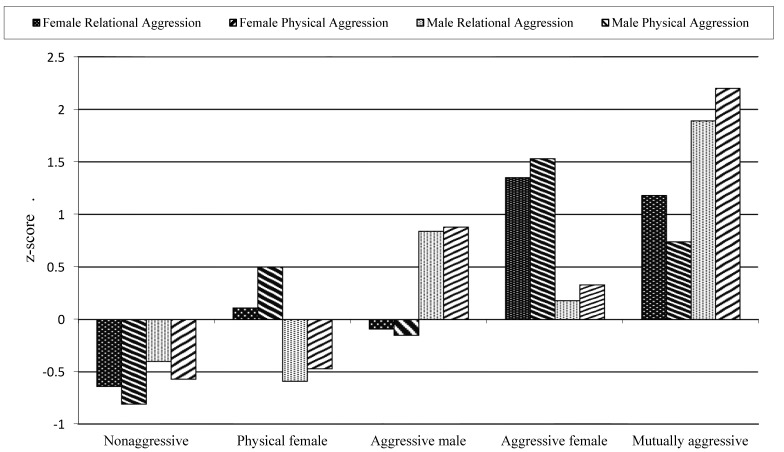
Standardized scores of the five romantic partner groups based on females’ and males’ reports of psychological and physical aggression.

The cluster analysis revealed that the five-cluster solution produced distinctive and homogeneous groupings that explained 62.9% of the error sum of squares. The standardized scores for these five clusters are presented in Figure 1. To interpret the clusters, we used ±0.5 *SD* as an indication of differences. The first group, termed *nonaggressive*, consisted of 79 dyads in which the males and females both reported below average levels of psychological and physical aggression. The second group, termed *physical female*, consisted of 38 dyads in which the females reported above average levels of physical aggression and the males reported low levels of aggression. The third group, termed *aggressive male*, consisted of 27 dyads in which males reported high levels of both psychological and physical aggression. The fourth group, *aggressive female*, included 34 dyads in which the females reported high levels of psychological and physical aggression. The final group, labeled *mutually aggressive*, consisted of 11 dyads in which both females and males reported high levels of psychological and physical aggression.

## 7. Differences between Romantic Relationship Groups

Chi-square analyses were performed to examine whether the five aggression subgroups differed in terms of demographic variables. Specifically, the chi-square analyses examined whether the five aggression groups differed with respect to ethnic composition (two Germans, one German and one immigrant, or two immigrants), duration of the relationship (less than four months, four to 12 months, or more than 12 months), and the participant’s household structure (intact or non-intact family). The chi-square test statistic was non-significant in all three of these analyses, indicating that the aggression groups did not differ with respect to these demographic measures.

A series of repeated measures ANOVAs were performed to examine whether the five aggression subgroups differed with respect to relationship functioning measures (see Table 3). In each of these analyses, cluster membership was included as the between-subjects factor, and male and female relationship functioning scores were included as the repeated dependent measure. For relationship perceptions, the five clusters did not differ with respect to reports of romance, but the cluster by reporter interaction was statistically significant for affiliation, *F*(4184) = 4.04, *p* = 0.004. Higher levels of affiliation were reported by females in the nonaggressive dyads than by those in the aggressive male and mutually aggressive dyads. Males in the nonaggressive and physically aggressive female dyads reported more affiliation than did those in the aggressive female and mutually aggressive dyad. For conflict prevalence, the mutually aggressive, aggressive male, and aggressive female dyads reported more frequent conflicts than nonaggressive dyads did. For jealousy, the interaction between cluster and reporter was statistically significant, *F*(4184) = 3.28, *p* = 0.013. Females in the five clusters did not significantly differ; males in the aggressive male dyads reported more jealousy than those in nonaggressive dyads.

**Table 3:** Mean-Level Differences in Females’ and Males’ Reports of Relationship Functioning as a Function of Romantic Partner Group.

	Romantic Partner Groups Non-Aggressive Physical Female Aggressive Male Aggressive Female Mutual					
	(*n* = 79)	(*n* = 38)	(*n* = 27)	(*n* = 34)	(*n* = 11)	*F*(4,184)
**Conflict**						
Prevalence	3.06_c_	3.16_bc_	4.30_ab_	4.12_ab_	5.78_a_	8.69 **
**Jealousy**						
Female	3.92	3.91	3.89	3.54	4.16	1.49
Male	3.33_b_	3.61_ab_	4.00_a_	3.59_ab_	3.79_ab_	2.45 *
**Relationship perceptions**						
Affiliation						
Female	3.46_a_	3.40_ab_	3.23_bc_	3.41_ab_	3.02_c_	5.83 **
Male	3.41_a_	3.47_a_	3.34_ab_	3.19_b_	2.86_b_	8.70 **
Romance	3.23	3.19	3.14	3.13	3.12	0.69

Note: Across rows, means with different subscripts differ significantly at *p* < 0.05 in Bonferroni comparisons. Male and female reports are presented separately for analyses in which relationship functioning differed as a function of romantic group; * *p* < 0.05; ** *p* < 0.01.

## 8. Discussion

Romantic relationships are new relationships which are experienced with much emotional intensity and variability. This study explored the different types of psychological and physical aggression in mid adolescent romantic couples from the perspective of assortative mating; it expands on existing knowledge by using dyadic data. Cluster analysis identified five different types of romantic relationships based on males’ and females’ reports of psychological and physical aggression that were meaningfully related to concurrent relationship functioning. The findings show that the similarity paradigm could not fully explain the diversity of types of aggression in adolescents’ romantic relationships. In contrast, concurrent relationship quality, an important, yet understudied variable shows meaningful links with aggressive behavior in romantic couples.

## 9. Types of Physical and Psychological Aggression in Adolescent Couples

Aggression in adolescent relationships is of major concern in many studies, due to relative stability over time within adolescents’ romantic relationships [3] as well as in adults’ marital relationships [4], and its potential impact for displaying antisocial behavior [14]. However, the overall levels in non-clinical samples were low. The participants in our German sample reported low to moderate levels of physical and psychological aggression, comparable with other published data on adolescents in North America and Europe [17,23,27,34], which provides support for the potential generalizability of our results. Also, and in accordance with the literature [48,49], females reported higher levels of psychological and physical aggression than males did.

In our analyses of theories that might explain why some dyads were aggressive and why some were not, we followed the “similar partner” theory [16], which proposes that aggressive adolescents are more likely to have a romantic relationship with similarly aggressive adolescents, or, conversely, that nonaggressive adolescents are more likely to pair up with similarly nonaggressive partners, a phenomenon which has been referred to as assortative partnering In fact, 48% of all couples followed such an “similarity attracts” paradigm, e.g. individuals select partners with similar characteristics. Most of them, 41%, were dyads in which both males and females reported below average levels of psychological and physical aggression. This seems to suggest that aggressive interactions were generally rare in these dyads and that the couple managed tensions in the relationship without any escalation. Only a small proportion of dyads (7%) were characterized by reciprocal aggression. Thus, it seems quite unlikely that aggressive adolescents form romantic relationships with partners who are similarly aggressive, at least for longer periods of time, as such relationships are normally prone to dissolution [39].

Although small in number, it is disquieting that we identified couples in which both male and female partners exhibited mutual physical and psychological aggression, and that these relationships last as long as relationships in the other dyads. Other studies have also identified profiles where both partners perpetrate and sustain aggressive interactions [50]. There is a well-replicated association between romantic partners’ level of antisocial behavior and an individuals’ antisocial level (see, for example, [51]). Further, as the findings of Monahan *et al.* [14] show, having a male partner elevated the chances of entering or remaining on a trajectory of antisocial behavior for females. Although we cannot, in our study, make any suggestions concerning the detrimental effects of reciprocal aggression for future antisocial behavior, these couples can be considered at risk and are in need of intervention before dating aggression turns into severe violence.

Similar to our findings, Espelage and Holt [52] found that 6% of bullying victims exhibited dating violence; other studies have reported an even higher percentage, up to 45%, of adolescents reporting being both the recipient and perpetrator of aggression [19,53]. Longitudinal studies on romantic aggression seem to suggest that such an aggressive interaction style does not necessarily persist [48]. Moffitt [54] has also reported that adolescent-specific antisocial behaviors typically decline around the age of 16 or 17 years. Nevertheless, there is also evidence that being in a relationship with an aggressive partner increases the odds of criminal behavior in young adulthood for both males and females [18]. Of note, in our study, the rates of male physical aggression were double those for female physical aggression. Thus, although mutual aggression characterized these dyads’ relationships overall, males reported more severe physical and psychological perpetration than their female partners did. It should be noted that all forms of aggression have a strong impact on well-being for the victimized [21].

A substantial proportion of adolescent couples, however, reported unilateral aggression (52%). One subgroup of aggressive males (20%) reporting moderate physical and psychological aggression is reminiscent to stereotypes of male dominance and female submissiveness [18]. Our finding could also be interpreted as reflecting a pattern of female self-silencing behavior (*i.e.*, when females consciously choose to remain silent in the wake of male aggression in order to preserve the relationship [55].

Notable was the relatively high proportion of females in our study with an aggressive interaction style that was not countered with aggression by their male partners. Such one-sided aggressive behavior, displayed by females when they were angry with or upset about their partners, emerged in two clusters. One cluster was characterized by dyads with female partners who showed average levels of physical aggression and male partners who showed very low levels of aggression (physically aggressive females, 20%). The other cluster was characterized by couples in which females reported high levels of both physical and psychological aggression, whereas their partners reported low levels in both types of aggression (physically and psychologically aggressive females, 17%). Male self-silencing as a pattern of dealing with female aggression has been consistently found among married and cohabiting adult couples [56], and according to our findings, seems to have an early onset. The health consequences of this behavior seem to be different for males and females; only female self-silencing is linked with depression [56].

Taken together, we found evidence for the dyadic similarity paradigm [16]; nonaggressive adolescents pair up with similar nonaggressive partners and only a small proportion of couples was found in which aggressive adolescents pair up with similarly aggressive partners. However, an even greater proportion of the adolescent couples did exhibit one-sided aggression with females being more frequently the aggressor than males. The analyses of other relationship qualities may shed some light on this issue.

## 10. Correlates of Relationship Functioning in Adolescent Couples with One-Sided and Mutual Aggression

When we analyzed relationship functioning, depending on cluster type, we found that adolescent couples in which both partners exhibited a nonaggressive interaction style showed the most adaptive pattern. Their relationships showed an equal balance between affiliative and romantic qualities (involving trust, friendship, sexual attraction, and desire for union), pointing to a high quality according to Seiffge-Krenke and Burk [30]. Despite these positive relationship features, jealousy and conflict prevalence in these couples was also quite high, reflecting the challenging nature of these early romantic encounters [29]. Apparently, both partners in these dyads were able to deal with tensions arising in the relationship and the way partners deal with tension and conflict seems to strengthen the relationship [31,57].

In the mutually aggressive dyads, conflict level was the highest, compared to other cluster types. In accordance with other studies, males in the mutually aggressive dyads were very jealous and controlling of their partner, which could lead to hostile patterns of communication and elevated conflict levels [3,24]. These findings are consistent with studies on distressed married couples, who report having more negative, aggressive interactions and lacking the capacity to effectively manage conflicts [1,4,58]. Of note, females and males in the mutually aggressive dyads experienced a low level of affiliative qualities (e.g., they lacked trust and acceptance in their relationship). The lack of trust was reflected in the high scores for jealousy [42] and conflict, and may have promoted an ongoing cycle of conflict and further aggression [7,8]. Adolescents who lack acceptance and trust in their relationship have fewer attentional resources at their disposal for managing conflicts [59]. This could be one reason for the escalation of conflicts in these mutually aggressive dyads.

Different theories might explain why some dyads were aggressive and why some were not. We mentioned already that the “similar partner” theory [16] accounts for findings in two clusters, the mutually aggressive dyads and the nonaggressive couples. The “problematic youth” theory [18] maintains that adolescents who show aggressive behavior may form intimate relationships with other aggressive adolescents due to their failure to establish contact with nonaggressive partners; this theory seems especially adequate to explain the behavior of the group of mutually aggressive dyads in our study, as they show deficits in managing tension and conflict in the relationship. Both theories state that, over time, both partners shape and reinforce each other’s attributes. For aggressive partners who form romantic relationship with similarly aggressive partners, this may lead to more aggression in the partner dyad and has the potential to spill over into aggression and antisocial behavior outside the partnership [15]. For those dyads in which both partners show nonaggressive behaviors, this may lead to stabilization of adaptive behavior and thus contribute to good relationship functioning. Although our study’s cross-sectional design does not allow for any causal prediction, our findings of concurrent associations between mutually nonaggressive behavior and positive relationship functioning as well as between mutually aggressive behavior and negative relationship functioning encourage us to endorse these theories. However, our findings must be validated by future longitudinal research.

We find it interesting that relationship quality was quite different in couples that showed one-sided aggressive behavior. Females in physically and psychologically aggressive dyads reported a similar good, trusting relationship to adolescent couples with a non-aggressive interaction style. This might explain their aggressive interaction style, as they seemed to experience no fear that the relationship might dissolve. It is possible that adolescent girls are more likely than adolescent boys to behave aggressively towards their partners because of unequal power and differences in negotiation styles. Some researchers have suggested that females have a greater need to establish autonomy in the emerging romantic relationship [60], which may result in more physical and psychological aggression. Foshee *et al.* [48] reported that when indirect forms of negotiating and teasing do not work, females employ more coercive, physically aggressive strategies.

In our study, we found that in all of the dyads with aggressive females, irrespective of whether they were both psychologically and physically aggressive or only psychologically aggressive, male partners did not respond with aggression. This points to gender-specific functions and interpretations of aggression [20]. Research on the double standards of aggressive behavior suggests that whereas aggressive boys are criticized by others, girls’ aggression towards boys is rarely taken seriously [61]. Whereas female aggression is viewed as defensive, male aggression is viewed as abusive, which may result in a downplay of female aggressive behavior in romantic couples. Studies have shown that boys and girls use aggression in romantic relationships for different reasons. Males often report that they show aggression in their romantic relationships when they are angry, whereas females report using aggression in self-defense [20]. Further, males may stay in a relationship because of sexual attraction and to maintain status in the peer group, even if the quality of the relationship is poor [62,63]. Thus, for males, balancing their affective or sexual needs or gain a more mature status might be an important reason why they pair up with a psychologically or physically aggressive girl [36,64]. Males may forgo responding to conflicts by showing aggression in order to calm their girlfriends down and not further exacerbate the conflict. This kind of male self-silencing behavior (“she will ignore me until I give in”) seems to be quite common [64]. In this regard, it is important to note the overall relationship quality in all dyads with aggressive females in our study was quite positive, suggesting that the one-sided aggression of females had not resulted in major damage to the relationship.

## 11. Limitations and Future Directions

Similar to other studies investigating adolescent couples [7,8], not all of the recruited participants reported having a romantic relationship were included in our analyses. We excluded couples whose relationships were very brief or in which one partner did not consider the relationship to be serious enough to participate with the other partner in the study. Thus, a replication of this study would be important, as the self-selective nature of the sample restricts the generalizability of our findings. As well, it would be important to replicate the study on a more ethnically diverse sample, as beliefs, expectations, and judgments about psychological and physical aggression vary with culture [65]. In addition, even though sexual experiences represent a crucial component of adolescents romance [66] and are likely to be intertwined in the link between romantic relationship and aggression, we did not directly assess the participants’ sexual activities. Due to the cross-sectional nature of our study’s design, we cannot be certain whether the negative relationship qualities found in our sample had a bearing on aggressive interactions or whether the aggressive interactions resulted in a poor romantic quality. As dating violence has been linked to a number of health consequences, including depression, alcohol and substance use, and low well-being [53,64], and has also the potential for antisocial behavior [14], the significance of these detrimental behaviors warrant continued study of the long- and short-term effects of aggression in romantic relationships. Future research will also need to examine how characteristics of interactions in past relationships are carried over into new dyads [67] and how prior dating experiences and selection patterns significantly influence new relationships [24], especially with the danger of assortative mating of aggressive partners. In addition, the relationship between conflict prevalence, dysfunctional conflict resolution styles and break ups, even in the same relationships (on-off relationships) warrants investigation [8].

This study focuses on romantic relationships from the perspective of both partners. However, a growing body of research is beginning has shown that family functioning predicts later romantic functioning [38,68]. Thus, future studies should also explore other factors such as family discord, ethnic minority status, and socioeconomic disadvantages [17,61], as aggression in romantic relationships is typically the end result of multiple negative societal and individual circumstances. In addition, peer violence and delinquent behavior are strongly related to aggressive dating relationships [2]. Attitudes that support aggression as a justifiable solution to conflict among couples have often been linked to reports of dating aggression [20] and future studies should therefore take this into consideration.

## 12. Conclusions

Aggression in adolescent romantic relationships is a serious issue. However, this study shows that investigations of adolescents’ romantic aggression need to incorporate the broader developmental context and consideration of the function aggression has in this context. In this study, males’ and females’ use of physical and psychological aggression in their romantic relationships emerged as a strong correlate of conflict prevalence, jealousy, and other relationship qualities. Many couples exhibited a non-aggressive interaction style and only few couples showed mutually aggressive patterns. A large proportion in our sample consisted of dyads with one-sided aggressive profiles in which females were more aggressive than their male partners. The lack of aggressive responses of their male partners suggests a gender-specific pattern in the evaluation and application of aggression as a way of resolving relationship conflicts. For the mutually aggressive dyads, adolescence is an important window of opportunity for intervention in these at-risk couples.
